# Oral aniracetam treatment in C57BL/6J mice without pre-existing cognitive dysfunction reveals no changes in learning, memory, anxiety or stereotypy

**DOI:** 10.12688/f1000research.11023.3

**Published:** 2018-06-22

**Authors:** Conner D. Reynolds, Taylor S. Jefferson, Meagan Volquardsen, Ashvini Pandian, Gregory D. Smith, Andrew J. Holley, Joaquin N. Lugo

**Affiliations:** 1 Department of Psychology and Neuroscience, Baylor University, Waco, Texas, USA; 2 Texas College of Osteopathic Medicine, University of North Texas Health Science Center, Fort Worth, Texas, USA; 3 Institute of Biomedical Studies, Baylor University, Waco, Texas, USA

**Keywords:** nootropic, aniracetam, learning, memory

## Abstract

**Background**: The piracetam analog, aniracetam, has recently received attention for its cognition enhancing potential, with minimal reported side effects.  Previous studies report the drug to be effective in both human and non-human models with pre-existing cognitive dysfunction, but few studies have evaluated its efficacy in healthy subjects. A previous study performed in our laboratory found no cognitive enhancing effects of oral aniracetam administration 1-hour prior to behavioral testing in naïve C57BL/6J mice.

**Methods**: The current study aims to further evaluate this drug by administration of aniracetam 30 minutes prior to testing in order to optimize any cognitive enhancing effects. In this study, all naïve C57BL/6J mice were tested in tasks of delayed fear conditioning, novel object recognition, rotarod, open field, elevated plus maze, and marble burying.

**Results**: Across all tasks, animals in the treatment group failed to show enhanced learning when compared to controls.

**Conclusions**: These results provide further evidence suggesting that aniracetam conveys no therapeutic benefit to subjects without pre-existing cognitive dysfunction.

## Introduction

In the 1970s, pharmacologist Cornelius Giurgea coined the term *nootropics* to describe a novel group of compounds capable of enhancing cognitive processes, intersynaptic communication, and the exchange of information between cerebral hemispheres. These compounds can be divided into five primary categories: cholinergic agonists, psychostimulants, piracetam compounds, hormones & essential nutrients, and agonists of cerebral blood flow ^1^. Initial interest in these compounds was limited to reversing the cognitive impairments in subjects with neurological damage or age-related decline. This investigation led to the development of a variety of neuroenhancing compounds, showing promise for cognitive restoration following epilepsy ^2^, traumatic brain injury ^3^, cerebral vascular accident ^4^, Alzheimer’s disease ^5^, and dementia ^6^. Nootropics have also been investigated in the treatment of many neurodevelopmental disorders, such as autism ^7^, ADHD ^8^, and schizophrenia ^9^.

Recently, there has been increasing prevalence of nootropic use among otherwise healthy subjects aiming to enhance academic performance, particularly college populations. According to recent population-based studies, the overall incidence of non-medicinal prescription psychostimulant use within the college student population is 4.1%–10.8% over the past year, and 6.4%–19.6% during their lifetime ^10– 14^. However, misuse of these medications can be dangerous, as psychostimulant toxicity has been linked to cardiac dysrhythmia, myocardial infarction, psychosis, and sudden death ^15, 16^.

The piracetam analog, aniracetam, has recently received attention due to its potential for cognitive enhancement associated with minimal reported side effects ^17^. In previous studies, aniracetam has been shown to enhance excitatory post synaptic potentials ^18^, reduce glutamatergic receptor desensitization ^18^, increase excitatory post-synaptic current (EPSC) decay time ^19^, and augment long-term potentiation in the hippocampus ^20^. Although the definitive mechanism of this compound is unclear, some evidence suggests that it acts as a reversible positive allosteric modulator of AMPA receptors ^21^. In addition to its purported cognitive enhancement, it has also been investigated for its anxiolytic effects ^22^. Aniracetam has proven effective in both human ^23^ and non-human ^24– 29^ models of cognitive dysfunction. However, few studies have evaluated its efficacy in healthy subjects without cognitive impairment. In addition, we included a repetitive behavioral test to examine some of the possible side effects of aniracetam treatment. We used the repetitive task as a behavior that we did not expect to be altered by the drug.

In a previous study, our laboratory evaluated whether daily oral administration of aniracetam (50 mg/kg) 1-hour prior to testing could improve cognitive performance in naïve C57BL/6J mice ^30^. Through a series of behavioral tasks, we observed that aniracetam did not improve spatial learning, fear learning, or motor learning. Further investigation of aniracetam pharmacokinetics suggested that peak serum levels are achieved approximately 30 minutes following oral administration ^31^. In light of this evidence, the current study aims to further evaluate aniracetam’s effects by administering aniracetam 30 minutes prior to testing, in order to optimize any cognitive enhancing effects. If aniracetam is truly a cognitive enhancer, we hypothesized that treated mice would display significantly greater learning and memory compared to controls.

## Materials and methods

### Experimental design

Twenty-four C57BL/6J male mice were generated at Baylor University for use in this study. The strain was originally purchased from Jackson Labs and bred at Baylor University. All mice were independently housed in a vivarium, where environmental conditions were controlled to an ambient temperature of 22°C with 12-hour light/12-hour dark diurnal cycles. All mice were also given *ad libitum* access to food and water. No health concerns were found during the courses of the experiments in this study. There were no adverse effects on the mice during the studies, and every effort was made to ameliorate any discomfort.

**Figure 1.**
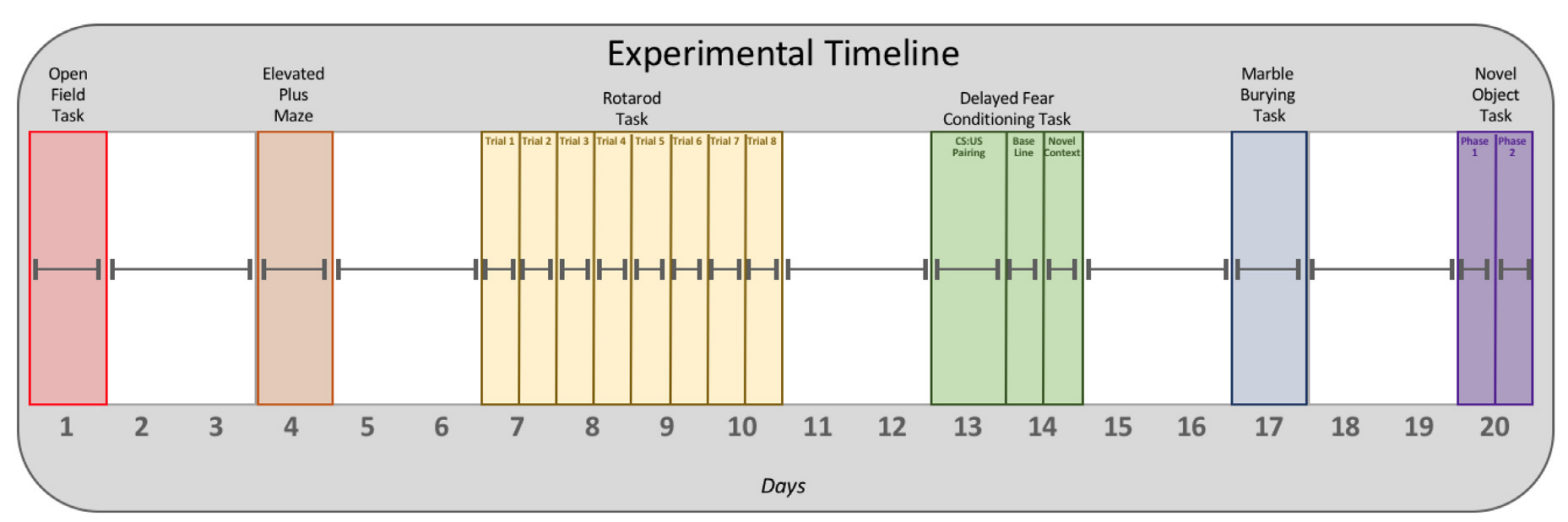
Experimental Timeline. The behavioral tests in this study were conducted in the following order: open field, elevated plus maze, rotarod, fear conditioning, marble burying, & novel object recognition. There was a period of 2–3 days of rest between in test in order to minimize the effect of repeated testing.

After reaching approximately 2 months of age, all mice were randomized to receive either one dose of aniracetam (100mg/kg) (1-[4-methoxybenzoyl)]-2-pyrrolidinone) (Shanghai Suyong Biotechnologies Inc., China), or an identical placebo by oral administration in a gelatin-based suspension 30 minutes prior to behavioral testing. Aniracetam or placebo was administered prior to each behavioral test. This route of administration was selected in order to mimic the typical mode of aniracetam consumption used in humans. During the double-blind phase, all mice were subjected to a battery of behavioral tests by designated experimenters blinded to treatment group assignments. An overview of the Experimental Timeline can be found in Figure 1. All behavioral testing was conducted during the middle seven hours of the light cycle to minimize time of day effects on performance. All procedures were conducted in compliance with Baylor University Institutional Animal Care and Use Committee, as well as the National Institute of Health Guidelines for the Care and Use of Laboratory Animals. All protocols were approved by the Baylor University Animal Care and Use Committee (Animal Assurance Number A3948-01).

### Fear conditioning

A two-day delayed fear conditioning protocol was used to assess amygdala-dependent learning. For this procedure, we used a 26cm × 22cm × 18cm operant chamber, composed of two clear acrylic sides and two metal sides, a metal grid floor capable of receiving an electric shock, an interior light providing constant luminescence (2 lux), and a speaker. The operant chamber was then placed inside of a sound attenuated isolation cubicle (Coulburn Instruments, Allentown, PA, USA) in order to control for external light and sound contamination. During all phases of this task, learning was assessed by the degree of freezing, as it is the most reliable measure of fear memory in mice. All testing was recorded and measured by automatic video tracking software, with visual confirmation of conditioned stimulus (CS) and unconditioned stimulus (US) presentations by the designated experimenter.

On the first day of testing, mice were placed into the operant chamber and 2 minutes of baseline activity levels were recorded. This was followed by a 30 second conditioned stimulus (CS) tone (80dB white noise), a 2 second unconditioned stimulus (US) shock (0.70 mA), and a 2-minute inter-trial interval (ITI). Another identical CS-US pairing was then presented and followed with a 30 second ITI.

The second day of testing consisted of two trials. During the first trial mice were placed back into the original operant chamber for 5 minutes and baseline activity was recorded. Before the second trial the operant chamber was modified with a foam pad under an acrylic square to cover the metal floor grid, an acrylic wall placed diagonally across that halved the space into triangular form, and 1mL of pure vanilla extract (Adam’s Extracts, USA) placed beneath the floor. These changes to the tactile, spatial, and olfactory contexts of the chamber were made in order to prevent context dependent learning. During the second trial mice were placed into the contextually modified operant chamber and 3 minutes of the trial baseline activity was monitored. This was followed by a 3-minute period of CS tone presentation (80dB white noise). All testing was recorded and measured by automatic video tracking software, with visual confirmation of CS and US presentations by the designated experimenter.

### Novel object

For this procedure, we used a 40cm × 40cm ×30cm clear acrylic open top box. This task was performed in an isolated room controlled for light levels, temperature, and background noise. Prior to testing, all mice were habituated to the arena without any objects for 20 minutes. During the first phase of testing, the two identical objects were placed on opposite sides of the apparatus and interactions with each were measured over a 10-minute period. During the second phase of testing, both objects were removed and replaced with the original object and a novel object. These were placed on opposite sides of the arena and interactions with each were measured over a 10-minute period. All trials were video recorded and manually scored by the designated experimenter after testing.

### Rotarod

The rotarod task was used to assess cerebellar motor coordination and learning. For this procedure, we used a rotating rod (Series 8 Rotarod; IITC Inc., Woodland Hills, CA, USA) which gradually accelerated from 5rpm to 40rpm. All mice were subjected to two 5 minute trials, with a 60 minute ITI, across 4 days of testing. The designated experimenter was responsible for monitoring and recording the length of time in which mice could hold onto the rotating rod before falling. This task was performed in an isolated room controlled for light levels, temperature, and background noise.

### Open field

The open field task was used to assess locomotion and anxiety. For this procedure, we used a 40cm × 40cm × 30cm clear acrylic box. This task was performed in an isolated room controlling for light levels, temperature, and background noise. All mice were placed in the center of the apparatus and allowed to explore for 10 minutes. Total time spent in the inner and outer regions were recorded and measured via Fusion optical recording system. Time spent in the outer and inner regions of the field was examined. A greater amount of time spent in the outer region is associated with anxious behavior.

### Elevated plus maze

The elevated plus maze task was used to assess levels of anxiety. For this procedure, we used a maze constructed of four white acrylic arms raised 40cm from the floor. All arms were 30cm long × 5cm wide. Two opposing arms were enclosed (walls, 15cm tall) and two opposing arms were left open. During this task, mice were placed in an open arm near the center of the maze and were allowed to explore for 10 minutes. Total distance and time spent in open versus closed arms was recorded by Noldus motion-tracking software (Ethovision, Netherlands). Video recordings were also manually scored by designated experimenters for additional behavioral observations, such as number of rearings in the open versus closed arms and number of head dips in the open arms. A greater amount of time spent in the closed arms versus open arms indicates higher levels of anxiety.

### Marble burying

The marble burying task was used to examine repetitive behavior. For this procedure clean home cages were filled with approximately 2–3cm of bedding and twenty black glass marbles were assembled into four evenly spaced columns of five rows. All mice were then placed into the testing cage in front of the array of marbles for 30 minutes. Several measurements of the percentage of the marble buried (50, 75, 100 and completely buried) was recorded by the designated experimenter. The measurement of 100% refers to a marble that is buried to its entire height with some bedding covering, but still in view of the experimenter, while completely buried marbles refers to those not in view of the experimenter. The increased marble burying reflects a higher tendency towards repetitive behavior.

### Statistical analysis

All behavioral data with a single measurement was analyzed using an independent samples t-test. The Independent samples t-test was used to analyze behavior in the open field, for day 2 of fear conditioning (fear memory), and for novel object recognition. All behavioral data with repeated measures were analyzed using a two-way Analysis of Variance (ANOVA), with experimental group as the independent factor and the trials or block number as the repeated factor. The two-way analyses were performed on rotarod data and on data from day 1 of fear conditioning (acquisition of fear learning). All data were analyzed using SPSS 20.0 (IBM, USA) or GraphPad Prism 7 software (La Jolla, CA). Values are shown as mean ± S.E.M. for each group.

## Results

### Aniracetam does not suppress anxiety levels or enhance overall activity

**Figure 2.**
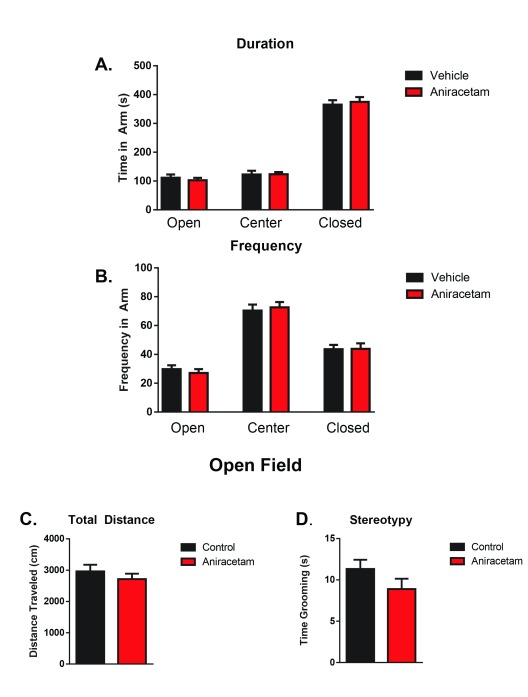
Aniracetam pretreatment does not change performance on the elevated plus maze or open field tasks. ( **A**) In the elevated plus maze test, an independent measures t-test revealed no significant differences in time spent in the open arms *t*(1,22) = 0.63, *p* = 0.53; center *t*(1,22) = 0.04, *p* = 0.23; or closed arms *t*(1,22) = 0.42, *p* = 0.67. ( **B**) There were also no significant differences in the number of entries into the open arms *t*(1,22) = 0.69, *p* = 0.49; center area *t*(1,22) = 0.39 *, p* = 0.69; or closed arms *t*(1,22) = 0.05, *p* = 0.96. ( **C**) In the open field test, an independent measures t-test revealed no significant differences between groups in total distance moved *t*(1,22) = 0.90, *p* = 0.37, ( **D**) or stereotypy time *t*(1,22) = 1.45, *p* = 0.16.

In the elevated plus maze task, we found no significant differences in time spent in the open *t*(1,22) = 0.63, *p* = 0.53; center *t*(1,22) = 0.04, *p* = 0.23; or closed arms *t*(1,22) = 0.42, *p* = 0.67 ( Figure 2A). Similar results were found in the frequency of arm entries, with no difference in number of arm entries into the open arms *t*(1,22) = 0.69, *p* = 0.49; center *t*(1,22) = 0.39, *p* = 0.69; or closed arms *t*(1,22) = 0.05, *p* = 0.96 ( Figure 2B).

In the open field task, we found no significant differences between the groups in total distance moved in the 10 minute trial *t*(1,22) = 0.90, *p* = 0.37 ( Figure 2C). There were also no significant differences observed in stereotypy time *t*(1,22) = 1.45, *p* = 0.16. ( Figure 2D) Together, these results suggest that aniracetam has no effect on locomotion or anxiety.

### Aniracetam does not enhance motor learning

**Figure 3.**
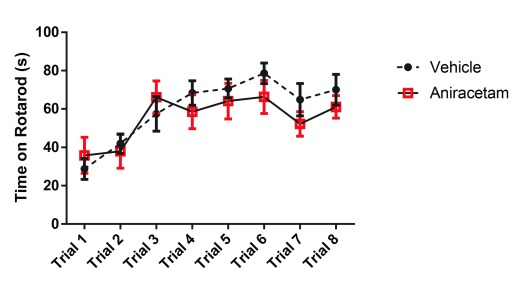
Aniracetam pretreatment does not change performance on the rotarod task. Across 8 trials, there was no main effect between groups ( *F* (1,22) = 0.4073, *p* = 0.5299). However, there was a main effect of learning across multiple trials ( *F* (7, 154) = 11.97; *p*<0.0001), indicating that motor learning had occurred within both groups.

Across 8 rotarod trials, we did not observe any main effect between groups ( *F* (1,22) = 0.4073, *p* = 0.5299) ( Figure 3). However, there was a main effect of learning across multiple trials ( *F* (7, 154) = 11.97; *p* < 0.0001), indicating that motor learning had occurred within both groups. These results suggest that aniracetam has no cognitive enhancing effect on motor learning.

### Aniracetam does not affect repetitive behavior

**Figure 4.**
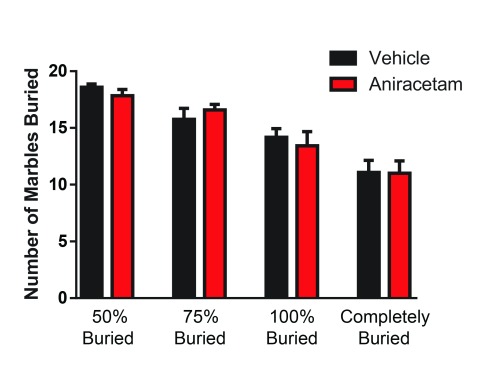
Aniracetam pretreatment does not change performance on the marble burying task. Independent measures t-tests revealed no significant differences in the animal’s performance in marble burying when measured at: 50% *t*(1,22) = 1.18, *p* = 0.24; 75% *t*(1,22) = 0.76, *p* = 0.45; 100% *t*(1,22) = 0.50, *p* = 0.61; or at the level of completely buried *t*(1,22) = 0.05, *p* = 0.95.

In the marble burying task, we found no significant differences in performance when measured at: 50% *t*(1,22) = 1.18, *p* = 0.24; 75% t(1,22) = 0.76, *p* = 0.45; 100% t(1,22) = 0.50, *p* = 0.61; or at the completely buried level *t*(1,22) = 0.05, *p* = 0.95 ( Figure 4). These results suggest that aniracetam has no effect on repetitive behavior.

### Aniracetam does not enhance associative fear memory

**Figure 5.**
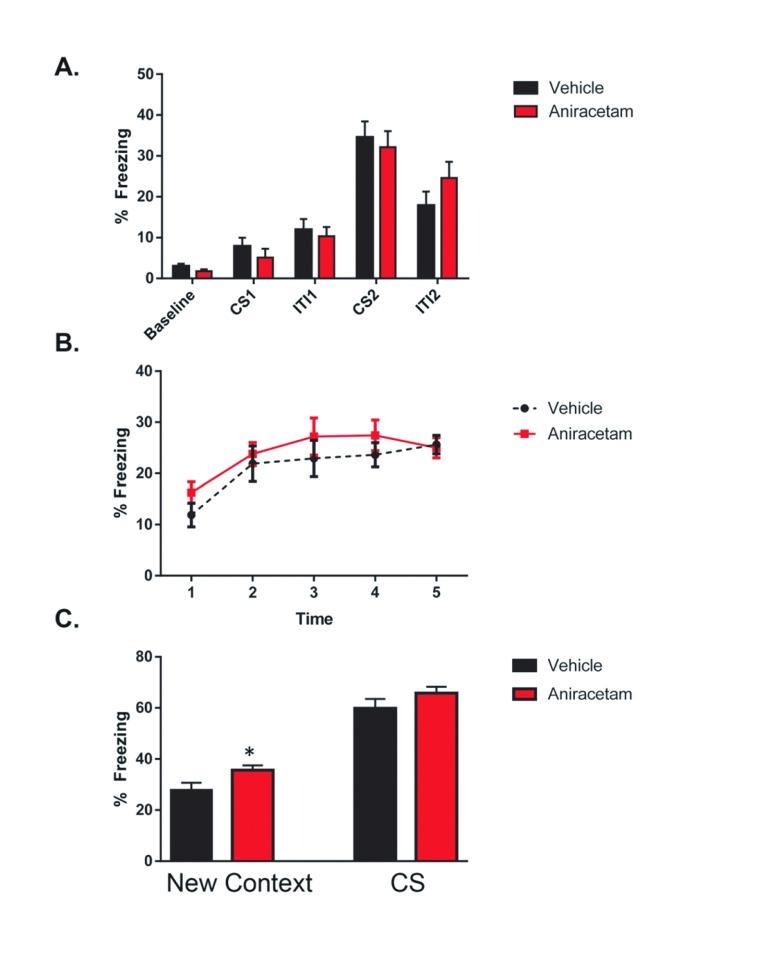
Aniracetam pretreatment does not change performance on the delayed fear conditioning task. ( **A**) On the first day of testing, mice were placed into the operant chamber and 2 minutes of baseline activity levels were recorded. This was followed by a 30 second conditioned stimulus (CS) tone (80dB white noise), a 2 second unconditioned stimulus (US) shock (0.85mA), and a 2-minute inter-trial interval (ITI). Another identical CS-US pairing was then presented and followed with a 30 second ITI. Following multiple foot shock and tone presentations, a two-way ANOVA test indicated no main effect of group (F (1, 19) = 0.1048; p = 0.7497) or interaction between groups (F (4, 76) = 0.5453; p = 0.7029). ( **B**) The second day of testing consisted of two trials. During the first trial, mice were placed back into the original operant chamber for 5 minutes and baseline activity was recorded. ( **C**) Before the second trial the operant chamber was modified with a foam pad under an acrylic square to cover the metal floor grid, an acrylic wall placed diagonally across that halved the space into triangular form, and 1mL of pure vanilla extract (Adam’s Extracts, USA) placed beneath the floor. These changes to the tactile, spatial, and olfactory contexts of the chamber were made in order to prevent context dependent learning. During the second trial, mice were placed into the contextually modified operant chamber and 3 minutes of the trial baseline activity was monitored. Aniracetam mice displayed significantly increased freezing in the novel context t(1,22) = 2.984, p = 0.004. This was followed by a 3-minute period of CS tone presentation (80dB white noise). There was no significant difference in the freezing behavior expressed between treated and control mice t(1,22) = 1.976, p = 0.052.

On the first day of fear conditioning, mice were placed into an operant chamber where multiple tone and foot shocks were administered. We observed no main effect of group ( *F* (1, 19) = 0.1048; *p* = 0.7497) or interaction between groups ( *F* (4, 76) = 0.5453; *p* = 0.7029) ( Figure 5A). However, there was a main effect of learning across multiple trials ( *F* (4, 76) = 42.35 *p* < 0.0001), indicating that fear learning had occurred within both groups. The second day of testing consisted of two trials. During the first trial, mice were placed back into the original operant chamber for 5 minutes and baseline activity was recorded. We observed a main effect of time F(4,120) = 6.363, p < 0.001, however there was no significant difference in freezing between groups F(1,120) = 2.546, p < 0.113 The second day of testing consisted of two trials. During the first trial, mice were placed back into the original operant chamber for 5 minutes and baseline activity was recorded. We observed a main effect of time F(4,120) = 6.363, p < 0.001, however there was no significant difference in freezing between groups F(1,120) = 2.546, p < 0.113 ( Figure 5B). During the second trial, mice were placed into the same operant conditioning chamber with novel context. For the first 3 minutes, mice were allowed to explore the novel context with no stimulus presentation. Aniracetam treated mice displayed significantly increased freezing in the novel context t(1,22) = 2.98, *p* < 0.01. Upon presentation of the tone both groups displayed increased fear, however there was no significant difference in the freezing behavior expressed between treated and control mice t(1,22) = 2.0; p < 0.05) ( Figure 5C). These results suggest that aniracetam treatment has no cognitive enhancing effect on associative fear learning.

### Aniracetam does not enhance novel object recognition

**Figure 6.**
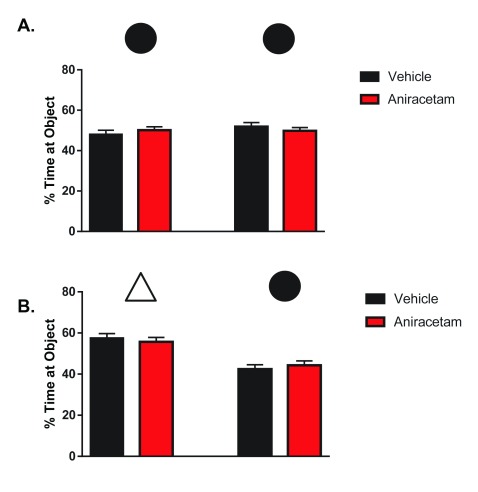
Aniracetam pretreatment does not change performance on the novel object recognition task. ( **A**) Independent measures t-tests indicated no significant preference towards the left or right object between treated *t*(1,22) = 1.333, *p* = 0.20 or control group mice *t*(1,22) = 0.1583, *p* = 0.88. ( **B**) An independent measures t-test indicated no differences in preference between groups in the novel object condition *t*(1,22) = 0.6112, *p* = 0.5474.

During the initial phase of testing, object preference was measured for identical objects. There was no significant preference towards the left or right object between treated *t*(1,22) = 1.333, *p* = 0.20 or control group mice *t*(1,22) = 0.1583, *p* = 0.88 ( Figure 6A). During the second phase of testing, object preference was measured between a familiar and novel object. There was a significant preference towards the novel object in both treated *t*(1,22) = 4.968, *p* < 0.0001 and control mice *t*(1,22) = 3.776, *p* < 0.001. However, there were no differences in preference between the groups in the novel object condition *t*(1,22) = 0.6112, *p* = 0.5474 ( Figure 6B). These results suggest that aniracetam treatment has no cognitive enhancing effect on novel object recognition.

Raw data for ‘Study of oral aniracetam in C57BL/6J mice without pre-existing cognitive impairments.’ (A) Open field total distance data and stereotypy results for vehicle and aniracetam-treated subjects. (B) Elevated-plus maze mean time and total frequency visits for open, closed, and center arms for vehicle and aniracetam-treated subjects. (C) Marble burying data for marbles buried at 50%, 75%, 100%, and total marbles for vehicle and aniracetam-treated subjects. (D) Delay fear conditioning data for day 1 and day 2 for vehicle and aniracetam-treated subjects. (E) Rotarod data for latency to fall off rotarod for vehicle and aniracetam-treated subjects. (F) Novel object recognition data for phase 1 and phase 2 for vehicle and aniracetam-treated subjects. Click here for additional data file. Copyright: © 2018 Reynolds CD et al. 2018 Data associated with the article are available under the terms of the Creative Commons Zero "No rights reserved" data waiver (CC0 1.0 Public domain dedication).

## Discussion

Although significant progress has been made towards understanding the neuroenhancing effects of aniracetam in subjects with cognitive impairment, there has been little investigation into its therapeutic effects on healthy subjects. In a previous study ^30^, our laboratory demonstrated that drug treatment in healthy C57BL/6J mice did not produce any significant effects on learning and memory, anxiety, locomotion, or repetitive behavior. Given the existing body of evidence supporting aniracetam’s cognitive enhancing effects, we elected to investigate this substrate further by using a modified drug treatment schedule to ensure peak serum levels during behavioral testing. Through this follow-up investigation, it was demonstrated that aniracetam conveys no significant cognition enhancing effects in healthy subjects.

Although aniracetam has a relatively short plasma elimination half-life (~30 min), long-term multiple administration is known to cause accumulation of its metabolites in the body ^32^. Several of these metabolites produce nootropic activity similar to their parent compound. One previous study demonstrated that administration of one metabolite, 2-pyrrolidinone, induced long-term potentiation of AMPA receptor responses in *Xenopus* oocytes ^33^. Given the pharmacokinetic properties of aniracetam and its accumulated metabolites, these would be expected to act synergistically to enhance cognition towards the end of behavioral testing. However, healthy mice still did not display any signs of cognitive enhancement.

Our findings are in contrast to a previous study by Rao *et al.* ^27^, which demonstrated that intrahippocampal aniracetam infusions significantly improved Y-maze performance in healthy rats. A key difference in experimental design between this study and ours is the route of administration. Intrahippocampal drug infusion provides tightly controlled, localized doses by circumventing first-pass metabolism. This method leads to a more accurate assessment of the drug serum levels necessary to achieve a therapeutic effect, but is restricted specifically to animal studies. Oral drug administration typically has a much lower bioavailablity due to hepatic biotransformation, but provides a higher ecological validity. For the purposes of our study, we elected to administer aniracetam orally, as it closely mimics the route of administration used most commonly in human reports. Although it is theoretically possible that cognitive enhancement could be achieved by aniracetam treatment, the therapeutic dose required to achieve this effect may be unrealistic.

One potential limitation of this study was the use of a single drug dosage. The approach we took in the current study was to use a single dose and use this dose across a number of behavioral tests. Although the 100 mg/kg dose used in this study is within the therapeutic range utilized by other studies ^24^, it is possible that a different dose would have produced significant effects. Future studies should aim to conduct behavioral assays with oral aniracetam supplementation across the full therapeutic range. One approach could be to use a dose range from 50 mg/kg to 1000 mg/kg and administer the dose before a fear conditioning test. If a cognitive enhancing effect is found through fear conditioning, then a more comprehensive series of behavioral tests could be used with the effective dose.

Despite any peer-reviewed data of non-medicinal use in humans, our findings contrast many subjective reports from healthy individuals purporting the cognitive enhancing effects of aniracetam and other piracetam-analogs. In a previous study, Corazza *et al*. ^34^ performed a multilingual qualitative assessment from a range of available online resources subjectively reporting benefits from piracetam use. These authors found that while the drug is used to improve academic and work-related performance, its use is also associated with side effects, such as hallucinations, dysphoria, fatigue, dizziness, memory loss, and headaches. Their findings indicate these side effects may be dose-dependent; however, because both the drug and their manufacturers are currently unregulated, it is impossible to determine an effective dose or therapeutic index in humans.

To our knowledge, both the present and previous studies from our lab represent the first empirical evidence of aniracetam treatment by oral administration in healthy subjects. Because this study closely mimics the drug administration in humans, we can infer that these results should most accurately depict the effects in healthy human subjects. Based on our findings, it can be suggested that non-medicinal and/or recreational use by healthy individuals may have only marginal therapeutic benefit, while the risk of harmful side effects remains.

## Data availability

The data referenced by this article are under copyright with the following copyright statement: Copyright: © 2018 Reynolds CD et al.

Data associated with the article are available under the terms of the Creative Commons Zero "No rights reserved" data waiver (CC0 1.0 Public domain dedication). 

**Dataset 1: Raw data for ‘Study of oral aniracetam in C57BL/6J mice without pre-existing cognitive impairments.’** (A) Open field total distance data and stereotypy results for vehicle and aniracetam-treated subjects. (B) Elevated-plus maze mean time and total frequency visits for open, closed, and center arms for vehicle and aniracetam-treated subjects. (C) Marble burying data for marbles buried at 50%, 75%, 100%, and total marbles for vehicle and aniracetam-treated subjects. (D) Delay fear conditioning data for day 1 and day 2 for vehicle and aniracetam-treated subjects. (E) Rotarod data for latency to fall off rotarod for vehicle and aniracetam-treated subjects. (F) Novel object recognition data for phase 1 and phase 2 for vehicle and aniracetam-treated subjects. DOI, 10.5256/f1000research.11023.d172542 ^35^
