# Supplementary material for: Oral aniracetam treatment in C57BL/6J mice without pre-existing cognitive dysfunction reveals no changes in learning, memory, anxiety or stereotypy
Source: F1000Res. 2018 Jun 22;6:1452. Originally published 2017 Aug 14. [Version 3] doi: 10.12688/f1000research.11023.3 (PMC5998011; doi:10.12688/f1000research.11023.3)
Supplement: Peer review history

# Peer review: Referee response for version 2

**DOI:** 10.5256/f1000research.14600.r34519

**Published:** 2018-06-12

**Authors:** Amy L. Brewster

**Affiliations:** 1 Department of Psychological Sciences, Weldon School of Biomedical Engineering, Purdue Institute for Integrative Neuroscience, Purdue University, West Lafayette, IN, USA

The study determined the effects of aniracetam, a cognitive enhancer, on anxiety and learning behaviors in normal adult mice. Aniracetam was administered orally at least 30 minutes prior to each behavioral test performed. This treatment paradigm did not produce significant differences between control-placebo and aniracetam- treated mice in the six behavioral tests evaluated. The authors conclude that acute treatment with aniracetam does not have cognitive enhancing effects in normal mice, when tested immediately after. 

 It would make it easier for the reader if a diagram with the experimental timeline is included in the manuscript. In addition, I would recommend that the authors discuss the observation that after several daily doses of aniracetam the animals still did not show enhanced cognitive abilities in the last behavioral tests performed. This is important because of a potential cumulative effect of the drug in the last tests performed.

I have read this submission. I believe that I have an appropriate level of expertise to confirm that it is of an acceptable scientific standard.

# Peer review: Referee response for version 2

**DOI:** 10.5256/f1000research.14600.r29078

**Published:** 2018-01-17

**Authors:** Marie-H Monfils, Laura Agee

**Affiliations:** 1 Department of Psychology, University of Texas at Austin, Austin, TX, USA

I am satisfied with the authors' changes to their manuscript.

We have read this submission. We believe that we have an appropriate level of expertise to confirm that it is of an acceptable scientific standard.

# Peer review: Referee response for version 1

**DOI:** 10.5256/f1000research.11890.r25058

**Published:** 2017-10-17

**Authors:** Stephan G. Anagnostaras

**Affiliations:** 1 Department of Psychology, University of California, San Diego, La Jolla, CA, USA

This is a fairly well-performed study but it has a serious flaw that isn't really addressed, which is that it uses a single dose. Single dose studies are problematic unless that dose is really well established as the appropriate dose, and it's really not very clear here how or why that dose is chosen. Although the dose chosen is maybe in the dose range people tend to take (maybe double the maximum dose people seem to report), pro-cognitive effects are notoriously dose-specific and it is possible they simply have the wrong dose. Rather than the very extensive behavioral approach, why didn't the authors simply run one task (e.g., fear conditioning) using a range of doses? At the very least the authors need to acknowledge this substantial limitation. 

 The other issues have been mentioned in the other review; in particular,  the fear conditioning parameters and scoring methodology are not adequately described.

I have read this submission. I believe that I have an appropriate level of expertise to confirm that it is of an acceptable scientific standard, however I have significant reservations, as outlined above.

# Response

**Published:** 2017-12-12

**Authors:** Joaquin Lugo

**Affiliations:** Baylor University, USA

We would like to thank Dr. Stephan G. Anagnostaras for his comments on our paper. We have included discussion on the limitation of the use of a single dose. We agree that this is a substantial limitation of our paper and the reviewer provided a great experimental design to determine an effective dose on cognition. We appreciate the input and plan to use this approach in future studies. We have also included more information on the methods of the fear conditioning test.

# Peer review: Referee response for version 1

**DOI:** 10.5256/f1000research.11890.r26005

**Published:** 2017-10-12

**Authors:** Marie-H Monfils, Laura Agee

**Affiliations:** 1 Department of Psychology, University of Texas at Austin, Austin, TX, USA

**Summary**

 The study was focused on assessing whether healthy (i.e. non-cognitively impaired) mice would display benefits following the administration of the nootropic drug aniracetam. Mice were run through a battery of tests aimed following oral administration of either a vehicle solution or an aniracetam solution. Though the main focus of the paper seemed to be on identifying whether aniracetam administered mice showed improvement in a variety of learning and memory tasks, mice were also assessed on measures of anxiety and repetitive behavior. The aniracetam treated mice did not show any significant improvements on any of the learning and memory tasks compared to controls. In addition, aniracetam treated mice did not display increased anxiety or repetitive behaviors as compared to control mice.

**Suggested Changes**

1. The title should be rewritten to include information about the outcome measures that are used. As it is, it is not readily clear from the title that this is a behavioral study.
2. The introduction does not provide an explanation for some of the tests that were run. Specifically, there was no mention of anxiety or repetitive behavior being linked with nootropic use. A discussion of this relationship should be included at some point in the introduction.
3. The authors do not mention which light phase the mice were run during. Given that all the outcome measures are behavioral and metabolic factors may influence how the mice process the aniracetam, this is a detail that needs to be included.
4. Whether or not the order of the tests was randomized and the amount of time that was given between each test should be mentioned.
5. The write-up of the fear conditioning methodology is missing a number of important details. The following need to be added: (1) A thorough description of the CS in use (e.g. was it a pure tone or white noise? What was the volume?), (2) the number of CS-US pairings, (3) the intensity and duration of the US, (4) the amount of time the mouse was given to habituate to the cage, and (5) the amount of time between CS presentations.
6. Excitatory post-synaptic current should be written out fully before being abbreviated to EPSC.
7. A description of Figure 4C needs to be added to the caption of Figure 4.
8. A significant difference between the freezing displayed by aniracetam treated rats and control rats is indicated in Figure 4B but the statistics backing up this finding are not included in the results or discussed at any point in the paper.

We have read this submission. We believe that we have an appropriate level of expertise to confirm that it is of an acceptable scientific standard, however we have significant reservations, as outlined above.

# Response

**Published:** 2017-12-15

**Authors:** Joaquin Lugo

**Affiliations:** Baylor University, USA

Dear Drs. Monfils and Agee,

We thank you for your input. We have revised the paper with your recommendations in mind. We believe the paper has been improved by their input. We have changed the title to reflect the behavioral outcome measures we used in the study. We have provided more justification in the introduction for the anxiety and repetitive behavior test we used in our experiments.

**Methods**

We included more information on the light cycle and when the mice were tested.

We included the order of the behavioral tests and more detailed information on the fear conditioning tests.

**Results**

We revised figure 4 to include figures 4A, 4B, and 4C. We also included all statistics for each portion of the tests. We wrote out a description of figure 4C in the caption.

**Discussion**

We wrote out the term excitatory post-synaptic current before we abbreviated it to EPSC.
